# Brilliant petawatt gamma-ray pulse generation in quantum electrodynamic laser-plasma interaction

**DOI:** 10.1038/srep45031

**Published:** 2017-03-24

**Authors:** H. X. Chang, B. Qiao, T. W. Huang, Z. Xu, C. T. Zhou, Y. Q. Gu, X. Q. Yan, M. Zepf, X. T. He

**Affiliations:** 1 Center for Applied Physics and Technology, HEDPS, State Key Laboratory of Nuclear Physics and Technology, and School of Physics, Peking University, Beijing, 100871, China; 2 Collaborative Innovation Center of IFSA (CICIFSA), Shanghai Jiao Tong University, Shanghai 200240, China; 3 Institute of Applied Physics and Computational Mathematics, Beijing 100094, China; 4 Science and Technology on Plasma Physics Laboratory, Mianyang 621900, China; 5 Department of Physics and Astronomy, Queen’s University Belfast, Belfast BT7 1NN, United Kingdom

## Abstract

We show a new resonance acceleration scheme for generating ultradense relativistic electron bunches in helical motions and hence emitting brilliant vortical *γ*-ray pulses in the quantum electrodynamic (QED) regime of circularly-polarized (CP) laser-plasma interactions. Here the combined effects of the radiation reaction recoil force and the self-generated magnetic fields result in not only trapping of a great amount of electrons in laser-produced plasma channel, but also significant broadening of the resonance bandwidth between laser frequency and that of electron betatron oscillation in the channel, which eventually leads to formation of the ultradense electron bunch under resonant helical motion in CP laser fields. Three-dimensional PIC simulations show that a brilliant *γ*-ray pulse with unprecedented power of 6.7 PW and peak brightness of 10^25^ photons*/*s*/*mm^2^*/*mrad^2^*/*0.1% BW (at 15 MeV) is emitted at laser intensity of 1.9 × 10^23^ W*/*cm^2^.

*γ*-ray is an electromagnetic radiation with extremely high frequency and high photon energy. As a promising radiation source, it has a broad range of applications in material science, nuclear physics, antimatter physics^1^,^2^,^3^, logistics for providing shipment security, medicine^4^ for sterilizing medical equipment and for treating some forms of cancer, e.g. gamma-knife surgery^5^. *γ*-ray from distant space can also provide insights into many astrophysical^6^,^7^ phenomena, including *γ*-ray bursts, cosmic ray acceleration at shock wave front, and emission from pulsar.

Generating intense bursts of high-energy radiation usually requires the construction of large and expensive particle accelerators^8^,^9^. Laser-driven accelerators offer a cheaper and smaller alternative, and they are now capable of generating bursts of *γ*-rays^10^. *γ*-ray generation has been demonstrated in a number of experiments on laser interactions with solid and gas targets, where the main mechanism is the Bremsstrahlung radiation of fast electrons interacting with high-Z material targets^11^,^12^,^13^,^14^,^15^,^16^,^17^. However, due to the small bremsstrahlung cross-section, the conversion efficiency of this scheme is rather low. Further, the broad divergence and large size of fast electron source also limit the achievable brightness of the generated *γ*-rays. *γ*-ray can also be produced by the nonlinear Compton backscattering, in which an electron beam accelerated by laser wakefields interacts with a counterpropagating laser pulse^18^,^19^,^20^,^21^,^22^. However, the number of electrons accelerated by laser wakefields in underdense plasmas is small, which also leads to rather low peak brightness of the produced *γ*-rays. Recent experiment^17^ demonstrates that the peak brightness of the *γ*-ray pulse at 15 MeV can reach only the order of 10^20^ photons/s/mm^2^/mrad^2^/0.1% BW.

With the progress of laser technology, laser intensities of 5 × 10^22^ W*/*cm^2^ are now available^23^ and are expected to reach the order of 10^23^–10^24^ W*/*cm^2^ in the next few years^24^, where the quantum electrodynamic (QED) effects play role in their interaction with plasmas. In the QED laser-plasma interaction regime, a promising mechanism for production of *γ*-ray photons is the nonlinear synchrotron radiation^25^,^26^,^27^,^28^,^29^,^31^ of ultrarelativistic electrons in the laser fields, i.e., 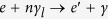
30. It is shown that *γ*-ray photons with the maximum energy extending to 100 s MeV can be generated by irradiating a solid target with an ultraintense laser^26^,^27^,^28^. However, for laser interaction with steep solid density targets, where no preplasmas exist, the *γ*-ray emission occurs only in the small skin-depth region^2^,^26^,^27^,^28^, therefore, the conversion efficiency from laser to *γ*-rays is still low, and the peak brightness of *γ*-rays is also limited. Recently, a self-matching resonance acceleration scheme^32^,^33^ in near-critical plasmas by circularly polarized laser pulses has been explored, which can generate much denser relativistic electron beams than the case of direct laser acceleration with linearly polarized lasers^34^,^35^. However, the laser intensity used there is comparatively low in the non-QED regime, electron resonance acceleration is dominantly governed by only the self-generated electromagnetic fields in the plasma, which limits both the energy and the density of the electron bunch for synchrotron radiation.

In this paper, by using a near-critical plasma interaction with ultraintense circularly polarized (CP) laser pulses, we report on a new resonance acceleration scheme in the QED regime for generating ultradense ultrarelativistic electron bunches in helical motions [see Fig. 1(a)] and therefore emitting brilliant vortical *γ*-ray pulses. In this QED scheme, on the one hand, because of the quantum radiation losses, the transverse phase space of electrons is confined^36^,^31^, and the electrons are easily trapped in the center of laser-produced plasma channel; on the other hand, due to the additional contribution of radiation reaction recoil force, the resonance bandwidth^37^ between laser frequency (in the electron rest frame) and that of electron betatron oscillation under quasistatic electromagnetic fields in the channel is significantly broadened, that is, the resonance condition is much relaxed. Both of these effects result in formation of an ultradense electron bunch under resonant helical acceleration in CP laser fields, where both the particle number and energy are much larger than those under only direct laser acceleration (DLA) by linearly polarized lasers^29^,^31^. Furthermore, the synchrotron radiation efficiency is much enhanced by the resonant electrons’ helical motion feature in the self-generated axial and azimuthal magnetic fields due to the use of CP lasers, comparing with that by linearly polarized lasers^29^,^31^, eventually leading to production of brilliant petawatt vortical *γ*-ray pulses. Three dimensional (3D) particle-in-cell (PIC) simulations show that brilliant *γ*-ray pulses with unprecedented peak brightness of 10^25^ photons/s/mm/mrad^2^/0.1% BW at 15 MeV and power of 6.7 PW are produced at laser intensity of 1.9 × 10^23^ W*/*cm^2^.

## Theoretical Analysis

The properties of *γ*-ray radiation depend strongly on electron dynamics. Let’ s start with the dynamics of a single electron interacting with the laser and self-generated electromagnetic fields in laser-produced plasma channel by taking into account of the QED effects. In the ultrarelativistic limit 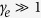, the radiation reaction force can be approximately written as^38^,^39^,^40^


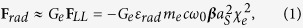


where **F**_*LL*_ is the classical radiation reaction force in the Landau-Lifshitz form^38^. In order to take the quantum effect of radiation reaction force into account, we use a quantum-mechanically corrected factor *G*_*e*_^39^,^40^, which reduces the amount of electrons’ unphysical energy loss due to the overestimation of the emitted photon energy in classical calculations. *ε*_*rad*_ = 4*πr*_*e*_/3*λ, r*_*e*_ = *e*^2^/*m*_*e*_*c*^2^ is the electron radius.***β*** = **v**/*c, a*_*S*_ = *eE*_*S*_/*m*_*e*_*ωc* corresponds to the QED critical field *E*_S_ (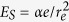), and 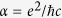 is the fine-structure constant. *e, m*_e_, **v** are electron charge, mass, and velocity, respectively, *ω*_0_ and *λ* refer to laser frequency and wavelength, and *c* is light speed. The probability of *γ*-photon emission by an electron is characterized by the relativistic gauge-invariant parameter 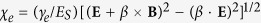, where *γ*_*e*_ is the Lorentz factor. The QED effects are negligible for 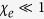 but play an important role for 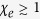.

Assuming a CP plane laser propagating along *x*-direction, the laser fields are *E*_*Ly*_ = *E*_*L*_ cos *ϕ, E*_*Lz*_ = *E*_*L*_ sin *ϕ, B*_*Ly*_ = −*E*_*Lz*_/*ν*_*ph*_, *B*_*Lz*_ = *E*_*Ly*_/*ν*_*ph*_, where *E*_*L*_ refers to their amplitude. The phase is *ϕ* = *kx* − *ω*_0_*t* and the phase velocity is *ν*_*ph*_ = *ω*_0_/*k*, where *ω*_0_ and *k* are laser frequency and wave number. The self-generated electromagnetic fields in the plasma channel are assumed to be *E*_*Sy*_, *E*_*Sz*_, *B*_*Sy*_, *B*_*Sz*_ transversely and *B*_*Sx*_ longitudinally. Considering the quantum radiation reaction force Eq. (1), the electron’s transverse motion in channel can be described as 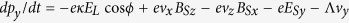, and 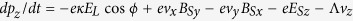, where κ = 1 − *ν*_*x*_/*ν*_*ph*_ and 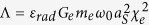. For high-energy electrons, it is reasonable to assume that 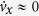, 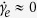 and 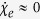, as they are slowly-varying comparing with the fast-varying *p*_*y*_ and *p*_*z*_. Further assuming 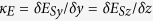, 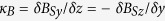, we obtain that


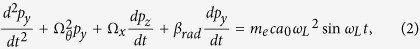



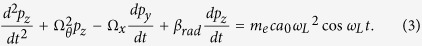


Here 
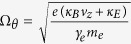
, 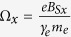 and 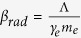. *ω*_*L*_ *=* *κω*_0_ refers to laser frequency in the electron rest frame, and *a*_0_ = *eE*_*L*_/*m*_*e*_*ω*_0_*c* is the normalized laser amplitude. In Eqs (2) and (3), the third term Ω_*x*_ is mainly distributed on the laser axis and can be neglected for the ultrarelativistic electrons in the electron bunch. Therefore, the betatron oscillation frequency can be estimated as 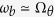. For electrons under resonance acceleration in laser fields, one can assume 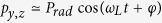, where *P*_*rad*_ is the momentum amplitude and *φ* is its initial phase. Substituting *p*_*y*,z_ into Eqs (2) and (3), one has


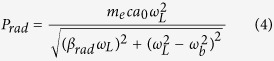


From Eq. (4), the amplitude of electron transverse oscillation can be obtained as


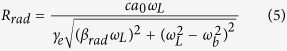


On the one hand, Eqs (4) and (5) show that *P*_*rad*_ and *R*_*rad*_ decrease when the radiation reaction factor *β*_*rad*_ increases, that is, the transverse phase space of the electrons is confined by the radiation reaction force^36^. This helps trapping of a great amount of electrons in the plasma channel center. On the other hand, by taking *dP*_*rad*_/*dω*_*L*_ = 0 from Eq. (4), one can get the resonance condition between laser frequency in the electron instantaneous rest frame and that of electron betatron oscillation *ω*_*r*_ in the plasma channel as


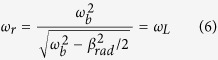


**Figure 1:**
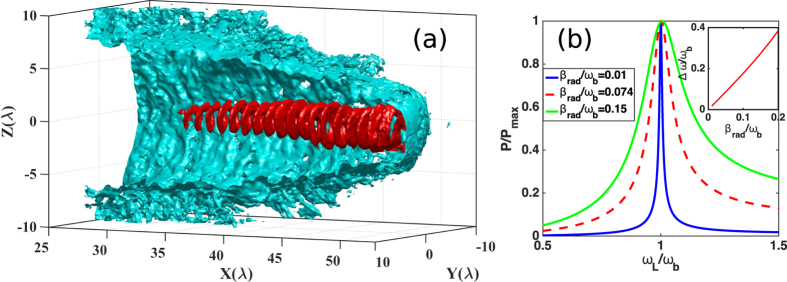
(**a**) 3D isosurface distributions for electron densities of the plasma channel (blue) and the helical electron bunch (red), where the isosurface values are respectively 30 *n*_*c*_ and 50*n*_*c*_; part (**b**) shows the resonance curve of the transverse momentum with *β*_*rad*_/*ω*_*b*_ = 0.01 (blue solid), 0.074 (red dash) and 0.15 (green solid); Inset of (**b**) shows the resonance bandwidth Δ*ω* with the radiation reaction factor *β*_*rad*_.

Here, one can treat Eq. (4) as a function of *P*_*rad*_(*ω*_*L*_), and the resonance curves for different radiation reaction factors *β*_*rad*_ are plotted in Fig. 1(b). It shows that the resonance bandwidth Δ*ω*, i.e., the full-width-at-half-maximum (FWHM) value of the resonance curve^37^, is significantly broadened when the radiation reaction factor *β*_*rad*_ increases [See inset figure of Fig. 1(b)]. Results indicate that the resonance condition of the accelerated electrons is much relaxed. Both of these effects eventually result in formation of an ultradense electron bunch under resonant helical acceleration by laser, and hence emission of unprecedented brilliant vortical *γ*-ray pulses.

## Simulation and Results

**Figure 2:**
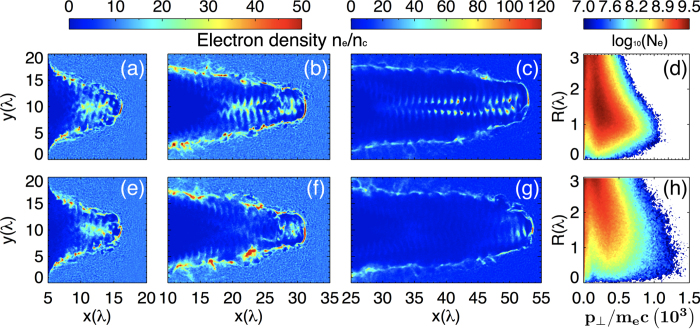
Density maps (in units of *n*_*c*_) of electrons in the plane z = 0 at *t* = 30*T*_0_ [(**a**) and (**e**)], 60*T*_0_ [(**b**) and (**f**)], 80*T*_0_ [(**c**) and (**g**)] for a CP laser pulse at intensity 1.9 × 10^23^ W*/*cm^2^ interaction with plasmas at densities 10*n*_*c*_ in the cases with (upper row) and without (lower row) the QED effects taken into account, respectively. (**d**) and (**h**) show the corresponding transverse phase space distributions of electrons at 80*T*_0_.

**Figure 3:**
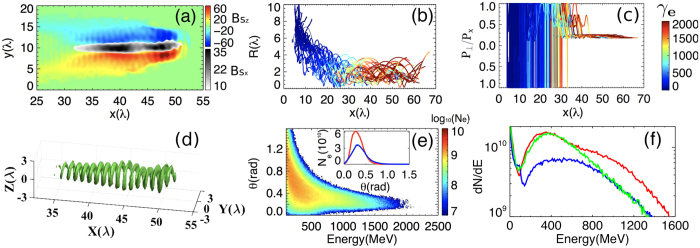
Properties of resonant electrons in the channel of Fig. 2: (**a**) self-generated magnetic field *B*_*Sz*_ and *B*_*Sx*_ (normalised by *m*_*e*_*ω*_0_/*e*) in the plane z = 0 at *t* = 80*T*_0_; (**b**) and (**c**) typical electron motion trajectories (
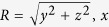
) and (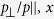), where the color bar shows increase of *γ*_*e*_ with time; (**d**) 3D isosurface distributions for electron energy density with the isosurface value at 1.2 × 10^4^*n*_*c*_*m*_e_*c*^2^; (**e**) the angular distribution of electrons; (**f**) the energy spectra of electrons in cases of with (red) and without (blue) the QED effects for CP laser and in the case of with QED effects for LP laser (green); Inset of (**e**) shows the distribution of the number of the electrons (energy above 500 MeV) with the polar angle.

To verify our scheme, 3D PIC simulations are carried out using the QED-PIC code EPOCH^41^, which takes into account of the QED effects in the synchrotron radiation of *γ*-rays by using a Monte Carlo algorithm^42^. From Fig. 1(a), we clearly see that an ultradense helical electron bunch is formed in laser-produced plasma channel, which undergoes resonance acceleration by the laser pulses. For comparison, simulations with the QED calculation switched off are also carried out. Figure 2 plots electron density maps in plane *z* = 0 at different times for the cases with (upper row) and without (lower row) the QED effects taken into account. At early time *t* = 30*T*_0_ [2(a) and 2(e)], both of the cases show similar characters that a number of electrons are firstly injected into the center of the plasma channel. However, at later time *t* = 60*T*_0_, they show completely different physics. For the case without the QED effects, most electrons in the channel do not satisfy the narrow resonance condition, and the focusing force provided by the self-generated electromagnetic fields is not strong enough to offset the laser radial ponderomotive force^31^, so that they are expelled from the plasma channel, as shown in Fig. 2(f). For the case with the QED effects, as predicted by our theory, the radiation reaction force provides not only “trapping” but also“resonant” effects on electrons in the plasma channel, where a great amount of electrons are trapped and undergo direct resonance acceleration by intense lasers, shown in Fig. 2(b). At *t* = 80*T*_0_, in the case with QED effects, the transverse phase space of electrons is adequately confined by the radiation reaction recoil force, shown in Fig. 2(d), compared with that in Fig. 2(h). We see from Fig. 2(c) that an ultradense relativistic electron bunch with density above 100*n*_*c*_ is formed in the channel under helical resonant motion in CP laser fields. Such an ultradense helical electron bunch leads to generation of strong axial magnetic field *B*_*Sx*_ up to 5.0 × 10^5^T and azimuthal B_*Sz*_ up to 8.0 × 10^5^T [See Fig. 3(a)]. The axial magnetic field helps to trap the background electrons near the laser axis undergoing pre-acceleration to hit the resonance condition^32^. The azimuthal magnetic field in turn not only provides additional confined forces to help trapping and achieving resonance acceleration of electrons, but also significantly enhances the probability of *γ*-photon emission^29^.

Figure 3(b) shows the typical electron motion trajectories under resonance acceleration. It can be seen that the electrons are injected from the front interaction surface and the wall of the plasma channel. As the role of quantum radiation losses increases, the transverse phase space of these electrons is confined^36^, and they are easily trapped in the plasma channel. Further with the aid of the radiation reaction force, as expected, a great amount of these electrons undergo resonance acceleration in CP laser fields, whose energies increase dramatically (see the color evolutions of the lines). For electrons undergoing resonance acceleration, their energies are gradually transferred from the transverse component into the longitudinal one by the **v** × **B** force, whose 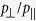 eventually decreases to a small value of 0.2, shown in Fig. 3(c). Figure 3(d) plots the energy density distribution of electrons at an isosurface value of 1.2 × 10^4^*n*_*c*_*m*_e_*c*^2^. It can be seen that an ultradense, helical relativistic electron bunch is formed, in which the electron maximum energy can reach 2 GeV [see 3(e) and 3(f)] and the total charge of electrons with energy above 500 MeV is about 200 nanoCoulomb [see the red line in 3(f)]. By comparing these high qualities of the beam with those without the QED effects, shown by the blue lines in Fig. 3(e) inset and 3(f), we conclude that the QED effects lead to great increase in the particle number and decrease in the divergence angle of high-energy electrons, in consistence with our theoretical expectations. The energy spectrum of electrons by using a LP laser with the same other parameters is shown by the green line in Fig. 3(f), which shows much lower cutoff energy and smaller number of high energy electrons, eventually leading to much weaker *γ*-ray emission.

**Figure 4:**
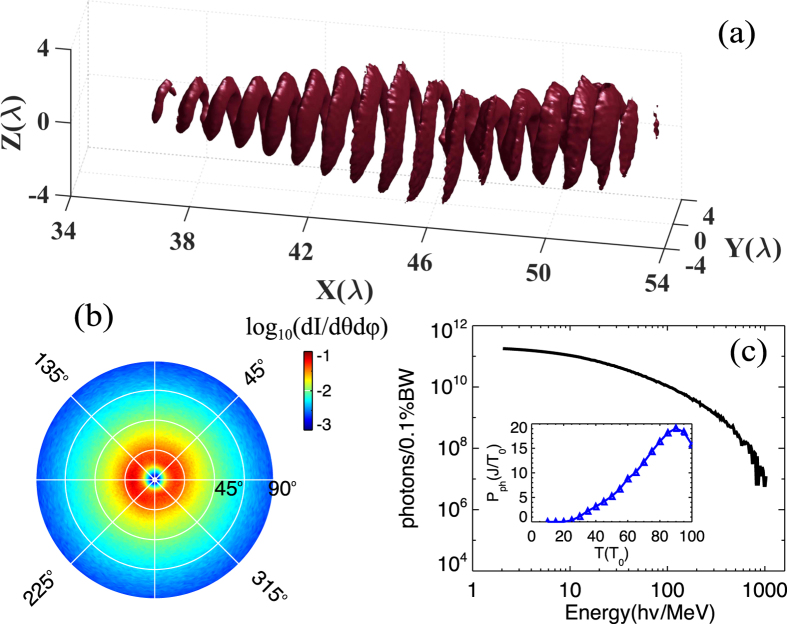
*γ*-ray pulse emitted in simulations: (**a**) 3D isosurface distribution of the *γ*-ray’s energy density at 5.0 × 10^3^*n*_*c*_*m*_e_*c*^2^; (**b**) the angular distribution of *γ*-ray energy for photons with energy above 2.0 MeV, where the polar and azimuthal angles are *θ* and *φ*, respectively; (**c**) the energy spectrum of photons, where the photon number is calculated in 0.1% bandwidth (BW); The inset in (**c**) shows the total radiation power *P*_*ph*_ = *W*_*ph*_/*T*_0_, which is defined as the emitted photon energy per laser period.

When the ultradense electron bunch undergoes resonance acceleration in CP laser fields, high-energy *γ* photons can be synchronously emitted. Figure 4(a) plots the 3D isosurface distribution of the *γ*-ray photon energy density at *t* = 80*T*_0_. It shows that a brilliant, vortical *γ*-ray pulse with energy density above 5.0 × 10^3^ *n*_*c*_*m*_*e*_*c*^2^, transverse size of 2 *μ*m and duration of 40 fs is generated. The radiation power can increase to 18 J/*T*_0_ (6.7 PW) and then decrease with the dissipation of the laser pulse [See the inset figure of Fig. 4(c)], more *γ* photons and higher energy conversion efficiency from laser to *γ*-ray can be obtained when the laser pulse is fully dissipated. At *t* = 100*T*_0_, the total number of *γ*-ray photons with energy above 2.0 MeV is about 3.08 × 10^14^ with a 15.9 MeV mean energy, and the total energy of *γ*-ray photons is about 780 J, which corresponds to 22.91% from the laser energy, significantly higher than that in the case using LP laser (16.15%). The energy spectrum of the *γ* photons at 80*T*_0_ is plotted in Fig. 4(c). The maximum photon energy exceeds 1.0 GeV, and the peak brightness of the *γ*-ray pulse at 15 MeV is 1.4 × 10^25^ photons/s/mm^2^/mrad^2^/0.1% BW. At *t* = 100*T*_0_, it can arrive at 3.5 × 10^25^ photons/s/mm^2^/mrad^2^/0.1% BW. To the best of our knowledge, this is the *γ*-ray source with the highest peak brightness in tens-MeV regime ever reported in the literature. From Fig. 4(b), we can also see that the high-energy photons are highly collimated.

## Discussion

**Figure 5:**
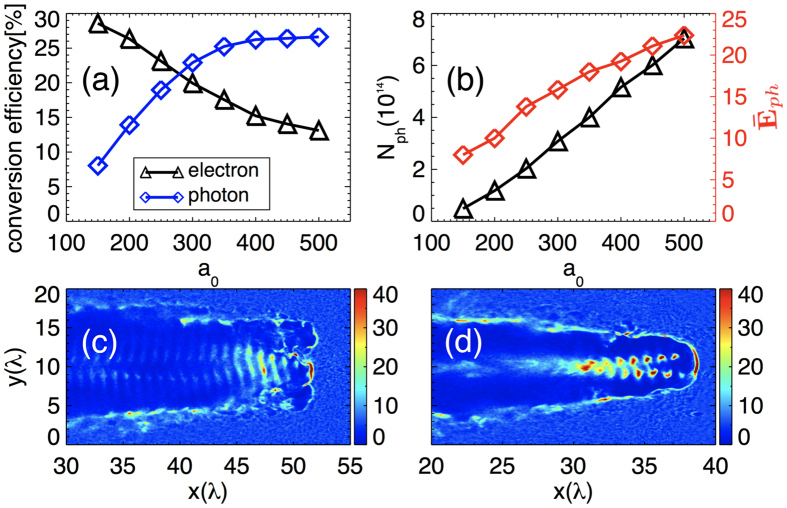
The scaling properties of the emitted *γ*-ray: (**a**) the conversion efficiencies from laser to electrons (black) and photons (blue) varying with different laser amplitude *a*_0_; (**b**) the photon number (black) and mean energy (red) varying with *a*_0_. (**c**) and (**d**) Respectively show the density maps (in units of *n*_*c*_) of electrons in the plane z = 0 for the case at normalized laser intensity of *a*_0_ = 150 and the case with a reasonable temporal profile of *a* = 250 sin^2^(*πt/*22*T*_0_).

The scaling properties of the emitted *γ*-rays by the proposed scheme have also been investigated. For a fixed self-similar parameter *S* = *n*_e_/*a*_0_*n*_*c*_ = 1/30, which strongly determines the electron dynamics in near-critical plasmas as discussed in ref. 43, a series of simulations are carried out with different laser intensities, i.e., *a*_0_. As shown in Fig. 5(c), our scheme still works at lower *a*_0_ = 150, though the density of the electron bunch drops, which leads to lower energy conversion efficiency of 8.0% from laser pulse to *γ* photons [Fig. 5(a)]. When the laser intensity increases, the energy conversion efficiency from laser to electrons drops and that from laser to photons grows up to 27% and then becomes saturate, shown by the blue lines in Fig. 5(a). And due to the enhanced resonance acceleration under stronger radiation reaction recoil force and self-generated electromagnetic fields, both the number and mean energy of the emitted *γ*-photons increase as well with the laser intensity, shown in Fig. 5(b). Different from the previous theoretical and numerical results 43, which is based on the betatron radiation properties in the non-QED regime with linearly polarized lasers (*a*_0_ ≤ 80), here the scaling shows to be as linear as *N*_*ph*_ ∝ *a*_0_ and 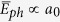 respectively.

To verify that our scheme still works for a more reasonable laser pulse, an additional simulation is performed. The laser pulse has a exact temporal profile of *a* = 250sin^2^(*πt*/22*T*_0_) with a duration of 11*T*_0_ (29.3 fs) and total energy of 266 J, which can be achieved, for example, with the ELI laser^24^ under development and Vulcan^44^ (planned updating) in the near future. The electron density map in plane *z* = 0 is plotted in Fig. 5(d). It can be clearly seen that an ultradense electron bunch is still formed undergoing stable resonance acceleration in the plasma channel and then brilliant *γ*-ray pulse is generated. As a result, 4.1 × 10^13^ *γ* photons are emitted and the energy conversion efficiency from laser pulse to *γ* photons can reach as high as 33%. Therefore, this robust electron acceleration and *γ*-ray emission scheme still works if a more reasonable laser pulse is used.

In this paper, we have reported a novel electron resonance acceleration scheme in the QED regime of CP laser-plasma interactions, where the quantum radiation loss helps trapping of electrons and the radiation reaction recoil force significantly relaxes the resonance condition between electrons and lasers. A great amount of electrons gather around the center of the plasma channel and undergo resonance acceleration, forming an ultradense, vortical relativistic electron bunch. As a result, unprecedentedly brilliant petawatt *γ*-ray pulses can be obtained.

## Methods

The 3D PIC simulations are carried out using the QED-PIC code EPOCH. In the simulations, 900 cells longitudinally along the x axis and 240^2^ cells transversely along y and z axes constitute a 75 × 20 × 20*λ*^3^ simulation box. A fully-ionized hydrogen plasma target with an uniform electron density of 1.7 × 10^22^ cm^−3^ (10*n*_*c*_) is located from *x* = 5 to 75 *λ*. Each cell of plasma is filled with 12 pseudoelectrons and 12 pseudoprotons. A CP laser pulse with peak intensity 1.9×10^23^ W*/*cm^2^ and wavelength *λ* = 0.8 *μ*m propagates from the left boundary into target. The laser pulse has a transverse Gaussian profile of FWHM radius *r*_0_ = 4*λ* and a square temporal profile of durations *τ* = 60*T*_0_ (*T*_0_ = 2*π/ω*_0_), which is composed of 30 *T*_0_ sinusoidal rising and 30 *T*_0_ constant parts.

The resonance curve of the transverse momentum in Fig. 1(b) is obtained by Eq. (4). The value *β*_*rad*_/*ω*_*b*_ = 0.074 is estimated from the parameters gotten in our simulation. For comparison in Fig. 3(f), under the premise of ensuring the same laser intensity, we have also carried out simulations that the incident laser is linearly polarized. The spectrums here are for electrons within a radius of 3 *λ* and a spreading angle of 0.38 rad. To investigate the scaling properties of the emitted *γ*-ray in Fig. 5, we fix the self-similar parameter *S* = 1/30 and the other parameters in the simulations are same. In order to check the accuracy of our simulation results, we have also carried out the simulation at a higher resolution with spatially half of the current grid size, which shows almost the same results as here.

## Additional Information

**How to cite this article:** Chang, H. X. *et al*. Brilliant petawatt gamma-ray pulse generation in quantum electrodynamic laser-plasma interaction. *Sci. Rep.* **7**, 45031; doi: 10.1038/srep45031 (2017).

**Publisher's note:** Springer Nature remains neutral with regard to jurisdictional claims in published maps and institutional affiliations.
